# Great Eras in Life

**Published:** 1893-06

**Authors:** W. C. Barrett

**Affiliations:** Buffalo, N. Y.


ARTICLE V.

### GREAT ERAS IN LIFE.

BY W. C. BARRETT, M. D., D. D. S., BUFFALO, N. Y.

**An address at the fifth annual dinner of the Chicago Odontological society.**

When the mariner leaves the shore familiar to him and ventures out upon an unknown sea, he marks his point of departure carefully, observes the time and position, and from that coigne of vantage all the events of the subsequent voyage are reckoned, until he again touches shore and finds a new landmark. All occurrences are dated from some epoch, whether it be in the history of nations or individuals. Wondrous incidents occur, which exercise a dominating influence upon generations yet to see the light, and they form points of departure for the voyage of a world through the great ocean of history.

The very day for this memorable meeting—aye, the special moment at which we assembled—was fixed and made definite by its remoteness from the time of the miraculous birth of the God child in the manger at Bethlehem. One thousand eight hundred and ninetythree years, nine days, six hours and thirty minutes from the time when the star of Bethlehem cast its era-making rays upon the lowly stable in that Jewish village, was the date that the managers of this feast set for our breaking of bread and tasting of the sacred salt of their hospitality.

Nations have their points of departure—memorable events in their history. England dates her regnant years from the time of the Norman conquest; from the day when the bold barons wrung the Magna Charta from an unwilling ruler; when Nelson broke through the French line at Trafalgar; when Victoria ascended the throne of Great Britain.

Nor is America without her great events. Her annals, though brief, comprise occurrences that have changed the history of the world. We reckon time from the Declaration of Independence; from the adoption of the Constitution; from the date of the Emancipation Proclamation. The first of these taught the world that States are by right self-formed, and not carved out by the sword. The second, that men have the right to govern themselves, and that there is no God-given privilege by which kings may assume power. The third forever settled and established human freedom.

It may be seen, then, that there are points of departure which are common to all mankind, and that there are others which are peculiar to single nations. So in man's personal history there are great events common to every one, and there are others that belong only to the individual. Let us for a moment consider some of those which affect the physical history of all men. They are great eras from which time may be reckoned. We may trace our progress in life by taking note of them. They are guiding-boards to point out the right way; light-houses fixed in the great current of time to warn us from the rocks and shoals that threaten shipwreck. He who heedfully studies their position and bearings may more confidently hope to glide out into the great open sea of eternity without having prematurely sunk his craft in the billows and eddies of time.

To enable one to recognize the events which may change the whole course of personal or national history, it is necessary that he study the indications that are always given. The Magicians of the East saw the star that heralded the natal hour of a Messiah, because they were watching by night. By the great mass of mankind the event passed unnoticed. When Napoleon dispersed the National Assembly, he was to all appearance but quelling a riotous outbreak. But it marked the era of Imperialism. The Rubicon was but a small stream, and when the legions of Cæsar forded its shallow waters it seemed but a trivial affair to them. But to Cæsar, who knew its significance, it meant either his entire destruction, or that he would be master in Rome, and sway the destinies of the civilized world.

The great physical eras of life are marked by unmistakable signs to him who is skilled in reading them. Nature never declines to reveal herself to the man who faithfully studies her methods. The changes which take place within the organisms are marked by changes without, and there is no era in the life of the individual that has not its clear and unerring visible token.

It is said that Death once promised an individual that his life should be prolonged for many years, and that three signs of his approach should be given him before the grim messenger called again. In the course of time Death again presented himself before the now aged man, and claimed him for his own. The man demurred, saying he had been promised a long life, but was answered: "Surely, fourscore years are the fulfillment of that pledge." "But," pleaded the old man, "I was promised three unmistakable signs of your approach." "They have been given," said Death, "and you might have seen them any day for the past year." "But for five years," said the man, my sight has been dimmed, so that I can see nothing distinctly."

"Then," Death answered, "you might have heard them whispered to you at morning and evening." "But," persisted the poor wretch, "my ears have lost their power to detect sound." "Then," said Death, "why have you not heeded the sign that has been given you every day at the table. You teeth have communicated the warning to you with every particle of food that you have put in the mouth." "But my teeth have long since left me, and my taste is so dulled that I cannot recognize it by that channel," said the man. "Wretch," answered Death, as he touched him with his paralyzing dart, "by your own admission you have long since received your promised warnings. Your eyes have become dim, your ears are dulled, your teeth have parted from you. What plainer warnings of my approach would you have ?

In watching for the turning points in our physical existence, there are no organs that tell the tale so unmistakably as those with which it is our special province to deal. They come with the morning of life, they leave at the sunset, and both their advance and retrogradation mark distinct periods in our life's history. At the opening of his course man is without them, because he has no use for them. His food must come to him already prepared. All his organs are feeble and undeveloped. He has not even sufficient strength to hold his head erect. His weak and helpless limbs refuse to bear his weight. He possesses little of coordinate muscular force. His digestive powers are as ineffectual as his muscular system. The pabulum that supports him must be received half-digested, and the mother's milk is alone adapted to his undeveloped stomach. After birth there is a period of rest; which lasts for a short time, when one of active growth succeeds it, for as in the natural world there are alternate periods of activity and rest—the spring, the summer and the winter —so in the physical world there are like times of advancement, and of consolidation of that which is formed.

Six months of slow solidification pass away, and the time arrives when the growing man enters into another period of existence. The utterly helpless, almost unconscious term is left behind, and the child begins to take note of its surroundings. The upper part of the body is so advanced that it can now hold its head erect. Its vision becomes distinct, and it begins to exercise muscular control. Its intellect shows signs of awakening, and it exercises some of the functions of voluntary life. This new arousing is not entirely a gradual one. It is comparatively a sudden, almost startling change, like that which takes place in the springtide of the year, when a few sunny days see a miracle wrought, the buds swelling and bursting, and all the floral world shaking out its plumage of green.

Is this stupendous change in the infant, this marvelous era in human existence, unmarked by outward manifestations? By no manner of means; for coincident with it the first teeth pierce the gums, an outward and visible token, indeed, of an inward and invisible change. The whole digestive apparatus has developed with the rest of the body. The child can now take nutriment that is a little more highly organized; and accordingly the mother's milk, which is its proper pabulum, assumes a new character. There is now more of the fat and less sugar. It would be utterly unfitted for the nourishment of a newly born child, for it has advanced in complexity with the growing organism. The first era of human life is completed, and it is marked by the presence of the first tooth.

Another period of rest and consolidation of that which is formed now succeeds, and the infant makes sure of the ground which it has gained. Six months more pass away, and another period of active growth succeeds, another springtime of development opens. The lower part of the body begins to grow. The brain assumes new functions. The digestive tract takes on fresh powers. The child gets a new stomach, and the mother's milk becomes yet more highly organized. The muscular system attains greater strength, and the helpless baby begins to help itself. A second era as distinct as the first has dawned, and the new development has the same index. The child has reached the age of twelve or fifteen months, and another dental organ is added to the four incisors already in place in each jaw. Helpless infancy is past, and the period of babyhood is gained. The change is a great one, but the era is not as marked as others of our existence. The food must still be prepared by the mother, and her milk is as yet the only proper nutriment.

Once more does nature demand a rest, and six months again pass away. Then comes another period of energetic development. There is a marked change in the character of the secretions. The saliva begins to contain ptyaline as an ingredient, and assumes a diastatic power. The function of converting starch into sugar is assumed, not in its full perfection, but as yet imperfectly. The villi of the intestines undergo a change in their action. The gastric juice has powers quite unknown to it before. New functions are awakened, and a distinct era in development is marked, as usual, by the appearance of another tooth. The child is now perhaps twenty months of age. It commences to care for itself. It walks; it perhaps speaks with intelligence. It exercises control over its excretory organs.

But the digestive functions are yet far from being complete. Its food must still be prepared for it. Its locomotion is yet uncertain, and the child is subjected to a thousand falls and mishaps, because of the immaturity of its muscular system. Attempts to feed it upon highly organized food result either in continued diarrhœas and febrile disturbances, or in a permanent weakness of the digestive system, and the growth of a confirmed dyspeptic. All is plainly indicated to him who intelligently watches the course of events. The deciduous denture is yet incomplete. Nature is but preparing, paving the way for a masticatory system. Up to the time of the advent of the latest tooth, none fit for grinding had yet appeared.

All that had previously been erupted were but cutters, incisors. It requires the whole apparatus for the perfect preparation of the food, and the denture was but half com-completed. The teeth that were most essential in trituration and insalivation were not in place, and this fact is indicative of a but partially completed era. The muscular system, the digestive tract, even the intelligence, were in the same imperfect, unfinished condition.. Their advance keeps pace with that of the teeth, and the latter are a sure index of the condition of the former.

But with the cutting of the first of the deciduous molars there is a marked change. As these teeth are the largest of all that have yet appeared, their advent marks a more momentous era. Function is now strengthened; new intelligences are awakened; the stomach has added tone and the power to transform into sugar the starches that form the greater portion of the human food is materially advanced. This tuberculous molar seems especially intended by nature for the comminution of solid food, and for its proper admixture with a saliva, for I believe the latter function the more important of the two. The diet of the child may now be safely changed. It can digest that which is not already partially digested by the mother. It is in most cases now fully weaned. It begins a more active existence. Its stomach increases in capacity, so that it will hold enough for a number of hours' nutritive purposes. That of the young infant suffices for but a short time. But development as a child is not yet complete, for it lacks something of a dull dentition.

Hence another period of slow development is a necessity, and then comes the last of the first teeth, and this marks the close of the period of infancy, and the commencement of childhood. The saliva has become a perfect fluid. The stomach is capable of digesting well-organized matter. The muscular system is sufficient for all the needs of the young child. The intelligence is so far awakened that it can begin to comprehend the simplest of abstract questions. The child is complete—as a child. That period of its existence is rounded out. It no longer depends upon the mother for the preparation of its food. From this time, growth is a more gradual process, and the periods between the development eras are materially lengthened. Heretofore we have found that there were intervals of about six months between the completion of periods. Now we reckon them by the same number of years.

The stage of infancy has indeed passed, but it is succeeded by another incomplete period. The young human being can walk with facility, but its strides are not those of a man. It can reason, but its mental processes are inductive: it has not arrived at the analytical, or even the synthetic stage. It can digest its food for itself, but that is mainly composed of the carbohydrates, and not of the albuminoid elements. At the end of the sixth year there appears the first of the permanent, as at the end of the sixth month there appeared the first of the deciduous teeth. This is the most important era in life, for now the great liability to these intestinal disorders, the falsely called diseases of dentition, has passed away; not because the cutting of teeth is over, but because the advent of this, the largest and most important of the whole dentition of man, marks the period when the digestive apparatus is so far developed and perfected that it can care for the highly organized food with which its infantile organs were unable to cope, but which too often was thrust upon them by ignorant and injudicious mothers and nurses. The terrible mortality of infancy is over. The child has escaped the deadly perils to which a majority of the human family succumb. Solid food is taken, is digested and assimilated, and the growth and consolidation of tissue goes on rapidly.

For another six years there is little change, save that which is incident to a period of steady, though comparatively slow, development. Nature is preparing for another grand effort, perhaps the most momentous of our existence. The deciduous teeth, which will soon be insufficient for the necessities of the man, are gradually and by imperceptible process removed, and larger and stronger ones take their places. Finally, this preliminary work all done, the proper preparations made, nature puts forth a supreme effort, and behold the opening of a new epoch.

Now, for the first time, sexual characteristics begin to appear. The male separates himself from the female, and begins to assume a new relation to her. The second molar appears. The period of childhood has passed; that of youth, of adolescence is at hand. The larynx is developed in the male, and the voice begins to change. In the female the breasts commence to swell. It is the beginning of the era of sexual development. There are in both sexes indescribable longings and undefined anticipations of the greatest change that takes place in human life. The child is an epicene no longer. Functions hitherto unknown are rapidly awakening. It is a period of unrest, of abnormal appetites, of occasional disturbed function, of unsatisfied yearnings. The desire for food is a constant craving, for the immature being is all animal. The newly awakened passions are undisciplined, and continually lead their possessor into strong adventures. There is no retrospect in the life that now is, but a persistent looking forward to the close of the era when the youth shall become a man, the girl a woman. It is the commencement of the period at whose close the Romans wisely ordained that the vesture should be changed, and the boy assume the manly toga.

Slowly this development continues. Gradually is sexual function progressed. The signs of virility in the male, and of sexual perfection in the female, are perfected and established. Another six years pass away, and behold the last of the teeth appear, and dentition is finally and fully completed. The last, most important period in man's developmental history is finished. Sexuality is complete. He is fully capable of procreating his species, and the female of bearing young. The human being has entered upon the last of the progressive eras of existence. There is no further growth; there is no more of the awakening of new functions, for the round is completed. Man and his dentition have reached their climax together. Thenceforth there is to be another long period of consolidation, of strengthening of that which formed, of active use of the intelligences now awakened, of the functions already born to man, of treading the highest summit of existence, and then begins decadence.

It is not my intention to follow the life history of man further. Development has ceased. The last grand era has been passed, and it was, like the others which we considered, marked by the advent of a tooth, that must necessarily be the last. I need pay no special attention to the fact that the progress of this growth in some manner depends upon environments; that these eras, especially the last, are hastened by a warm climate and retarded in the frozen north. That is true of all development. The general law is that when one function is early called into action, all the others are influenced by the same precocity. If the boy or the girl reaches puberty at an unusually early age, the other developmental eras have also been hastened. If the teeth are cut before the usual time, it is probable that the whole digestive tract is in a like advanced condition.

Undue stimulation may cause premature development of organs or sets of organs. These are not, however, evidences of strength, but rather indications of weakness. The intellect, through abnormal conditions, may be awakened too early, and the child may exhibit signs of mental power that should come only with the regular and coordinate growth of the rest of the system. Such precocity will not be accompanied by the usual indication of development in the advent of the teeth, because it is something apart from the regular processes of nature. Unless there be a steady progression, with the ripening of all the powers and the functions of the body together, it is an abnormal and unhealthy condition, and the lack of functional symmetry will surely end in a cutting short of the periods of healthy growth, and a final breaking down of the whole, from lack of sympathetic and harmonious action. If the teeth which should mark the advent of the epochs in man's life are empted prematurely, and in advance of the periods which they were properly destined to indicate, their existence will doubtless be brief, for the ripening of the other functions upon which their nutrition and consolidation depend is sufficient for their support. The teeth that are occasionally found in the mouth at birth are usually soon lost. The man only is the perfect man in whom there is an even balance of the functions and organs. Progression must be synchronous througbout. Undue prococity is as much a diseased condition as retardation. If it be confined to a single organ or set of organs, it means that the disturbed functional activity is due to some untimely stimulation, and that it is at the expense of other organs.

Call up from the misty past the visions of your own experience as dental surgeons, and inquire if they do not in the main corroborate that which I proclaim. Summon as witnesses the instances that you have yourselves observed, in which teeth were cut long before the stated period for their advent, or were unduly delayed after it, and learn if their possessor had a complete and equally developed structure, and if he preserved it to a healthy old age. Were there not neuroses, and an unbalanced mental condition? Was not the physical growth incomplete, the stature low and the muscular system feeble? If not, then has your experience been at cross-purpose with mine? It is only of him to whom the natural eras of life have come in their regular sequence that it can be said that his "age is as a lusty winter; frosty but kindly."

All biographical history but confirms that which I assert. Alexander the Great was said to have had teeth at his birth, and he was a drunkard and a neurotic. Cæsar was born with teeth, and Cæsar was an epileptic. Richard the Third had teeth thus prematurely developed; and he was humpbacked. Where are all the child-gen'uses that have astonished the world? Their flame of life burst forth with precocious brilliancy, but it soon went out in death, or early blight and decay of their phenomenal powers. Chatterton, "the marvelous boy, the sleepless soul that perished in its pride," the model of all precocious geniuses, also stands as their type in premature decay. The great eras of life must preserve their relative duration, must be marked by their accompanying outward signs, or the whole sequence of life is destroyed. An irregular dental development usually means an unbalanced organization, The healthy mind is found only in the healthy body. Who will not believe that Carlyle might have been capable of even greater things, had not his digestion been ruined by injudicious dieting in his youth, feeding with solid food before his teeth gave indications of the advancement of his digestive functions.

I would that I had time, and you the patience, to enter upon a consideration of the lessons which we as dentists may gather from a study of these questions. I should be delighted to speak to you about the so-called diseases of dentition, and to convince you, as I believe I could, of the fact that the cutting of the teeth has nothing whatever to do with the virulence of the disturbances that sweep away one-third of the human family before they have reached the age at which the deciduous teeth should all be in position. But I will spare you this, though I am strong in the faith that I could more benefit the human race by teaching this faithfully, than I could by preaching the most astounding dogma that ever religious council formulated.

It is *not* an exaggeration when we declare that the duties which fall to the dentist are among the most important of those entrusted to man. Disease and pain warp the intellect, and dwarf the understanding. He who allays suffering, works for the better development of the whole human race. "Canst thou not minister to a mind diseased?" asked Macbeth. "Ay," answers the modern dentist. "By my art can I quiet the disturbed organs, soothe deranged function, liberate the mind pent by pain, and bring harmony out of direst discord." In the performance of this holy task in which we are engaged, I can only exclaim with Tiny Tim. "God bless us every one." —*Dental Pract. and Advertiser.*

